# Sexual assault in the mahdia region : Epidemiological peculiarities of victims

**DOI:** 10.1192/j.eurpsy.2021.1460

**Published:** 2021-08-13

**Authors:** S. Brahim, M.A. Mosrati, W. Bouali, M. Kacem, S. Sellami, A. Abid, S. Ben Frej, M. Henia, M. Chabbouh, L. Zarrouk

**Affiliations:** 1 Psychiatry, University Hospital of Mahdia, Tunisia., chebba, Tunisia; 2 Legal Medecine, University Hospital of Mahdia, Tunisia., Mahdia, Tunisia; 3 Department Of Psychiatry, University Hospital Of Mahdia, Tunisia., Psychiatry, Mahdia, Tunisia; 4 Psychiatry, University Hospital of Mahdia, Tunisia., Mahdia, Tunisia; 5 Anesthesia, University Hospital of Mahdia, Tunisia., mahdia, Tunisia; 6 Psychiatry, University Hospital Of Mahdia., Mahdia, Tunisia

**Keywords:** epidemiology, sexual assault, rape, Molestation

## Abstract

**Introduction:**

Women around the world are still victims of violence and discrimination in many areas. In Tunisia, discrimination against women remains a reality, and they are often more vulnerable to violence, especially sexual violence, compared to men.

**Objectives:**

To describe the epidemiological characteristics of victims of sexual assault in the Mahdia region in Tunisia

**Methods:**

This is a descriptive and retrospective study of 110 sexual assault cases examined at the legal medecin department of Mahdia University Hospital between January 2016 and August 2018.

**Results:**

The majority of victims were female (80 %). All genders, 77% were under the age of 25 years old. The median age of the men was 11.5 years. The median age of women was 18. The urban origin was more common (55.5%). Only 8.1% were married compared to 87.4% single. Only 2.7% said they were divorced and only one woman was a widow. 41.8% of the sample said they were still in school and almost 29,1% of the cases were out of work. 3.6% reported a history of sexual assault. The sexual act was the same in all situations. The perpetrator was unique in 73.6% of cases, male (100%), known to his victim (57%) or even a member of the family circle (14%). Sexual assault by penetration was mostly reported (51%), and it was almost exclusively penile(98.2%).

**Conclusions:**

Sexual violence remains under-reported. The statistical data do not allow to know the phenomenon of its whole, because the majority of acts remain unknown, due to the absence of complaints or medical consultations.

